# Ocular and Orbital Involvement in Extranodal Natural Killer/T-cell Lymphoma: A Systematic Review of Clinical Outcomes and Prognostic Factors

**DOI:** 10.7759/cureus.109669

**Published:** 2026-05-26

**Authors:** Ramnik Kaur Buttar, Aphrodite Fotiadou, Vinay Patel, Alexandra Papoudou-Bai, Chris Kalogeropoulos, Panagiotis Kanavaros, Dimitrios Kalogeropoulos

**Affiliations:** 1 Department of Ophthalmology, Stoke Mandeville Hospital, Buckinghamshire Healthcare NHS Trust, Buckinghamshire, GBR; 2 Department of Anatomy/Histology/Embryology, Faculty of Medicine, School of Health Sciences, University of Ioannina, Ioannina, GRC; 3 Department of Radiology, Royal Berkshire Hospital, Royal Berkshire NHS Foundation Trust, Reading, GBR; 4 Department of Ophthalmology, Ruprecht Karls University of Heidelberg, Heidelberg, DEU; 5 Department of Ophthalmology, Faculty of Medicine, School of Health Sciences, University of Ioannina, Ioannina, GRC; 6 Department of Ophthalmology, Bristol Eye Hospital, University Hospitals Bristol NHS Foundation Trust, Bristol, GBR

**Keywords:** extranodal natural killer/t-cell lymphoma (enktl), natural killer/t-cell lymphoma, ocular lymphoma, orbital involvement, prognostic factors, systematic review

## Abstract

Extranodal natural killer/T-cell lymphoma (ENKTL) is a rare and aggressive malignancy, with ocular and orbital involvement representing an uncommon but clinically important presentation. Many cases are frequently misdiagnosed due to their ability to mimic common inflammatory and infectious eye conditions, resulting in delayed diagnosis and poorer clinical outcomes. This systematic review aimed to evaluate clinical outcomes and prognostic factors in ocular and orbital ENKTL. A PRISMA-compliant systematic review was registered with the International Prospective Register of Systematic Reviews (PROSPERO; CRD420261348194) and conducted using PubMed, MEDLINE, Embase, and Cochrane Library from database inception to 19 March 2026. Twenty-eight studies comprising approximately 429 patients were included. Data were extracted on patient demographics, treatment, survival, and prognostic factors, and analysed using a narrative synthesis. Median survival ranged from six to 17 months, with high mortality and frequent reports of rapid clinical deterioration. Delayed diagnosis, advanced disease, central nervous system involvement, and relapse were associated with poorer outcomes. Non-anthracycline, asparaginase-based regimens were associated with improved disease control compared with conventional therapies, although outcomes remained poor overall. Visual outcomes were similarly unfavourable. Improved survival was associated with early recognition, prompt biopsy, and timely initiation of appropriate treatment.

## Introduction and background

Ocular and orbital lymphomas are rare extranodal lymphomas that affect ocular and periocular structures. They can be classified into two main cell types: B-cell lymphomas, which include primary vitreoretinal lymphoma (PVRL), the most common type of intraocular lymphoma, strongly associated with primary central nervous system (CNS) lymphomas [1]; the second main cell type is extranodal natural killer/T-cell lymphoma (ENKTL). ENKTL is a rare and aggressive subtype of lymphoma strongly associated with Epstein- Barr virus (EBV), characterised by angiocentric and angiodestructive growth, primarily affecting the ocular adnexa [2]. It often involves the orbit and eyelid rather than being a true intraocular lesion, in contrast to B-cell lymphomas such as PVRL and mucosa-associated lymphoid tissue (MALT) lymphomas [3].

In this systematic review, we will focus on the clinical outcomes of ENKTL and how these are influenced by prognostic factors. It is important to note that the most common extranodal ENKTLs arise from the nasal cavity, nasopharynx and paranasal sinuses, with primary orbital cases exceedingly rare [4]. ENKTL is strongly linked to EBV, and in orbital disease, involvement is often due to secondary dissemination from the primary nasal tumour [5].

Although localised disease is potentially curable, symptoms are often insidious and misdiagnosed as common benign inflammatory eye conditions due to their non-specific nature [6]. Reported presentations often resemble uveitis [7], orbital inflammatory disease [8] or peri-orbital cellulitis [9], with patients most commonly reporting symptoms of visual impairment, generalised pain, and peri-orbital swelling. Due to the highly aggressive nature of this disease and clinical overlap, early diagnosis is often delayed, leading to a poor prognosis [10].

ENKTL is an aggressive disease, and prognosis is highly dependent on stage and tumour location. Poor prognostic factors include age >60 years, advanced disease (stage III/IV), lymph node involvement and EBV-DNA positivity. The prognostic index of natural killer lymphoma (PINK-E) is divided into three separate risk categories. The three-year survival rate is as follows: low risk (81%), intermediate risk (62%), and high risk (25%) [11].

The highest incidence of ENKTL is observed in East Asian populations, where ENKTL accounts for 7-10% of all non-Hodgkin lymphomas (NHLs). There is also a notably higher incidence in Latin American populations, with high rates in areas like Mexico and Peru, where the disease accounts for 5-7% of all NHLs. In European and North American countries, the incidence is comparatively low, and ENKTLs represent <1% of all NHLs. The reasoning behind this increased incidence is thought to be due to the higher prevalence of EBV and the genetic susceptibility to NHL in certain populations [12-14]. A study in Mexico by Quintanilla-Martínez et al. (1998) investigated the association between T-cell lymphomas and EBV. The study showed EBV infection in 70-90% of all T-cell lymphomas, a majority of which were of natural killer (NK)/T-cell lineage [15].

Due to the rarity of these tumours, many clinicians may never encounter this disease in practice and may not have the relevant clinical knowledge needed to diagnose and treat this condition with confidence. There remains a significant gap in the literature with limited studies systematically evaluating clinical outcomes and prognostic factors in ocular and orbital ENKTL. The current evidence is sparse, with many studies consisting of single case reports or small case series.

This systematic review aims to analyse and collate the current literature to provide a comprehensive evaluation of the clinical outcomes and prognostic factors associated with ocular and orbital involvement of ENKTL.

## Review

Methods

Our systematic review protocol was registered with the International Prospective Register of Systematic Reviews (PROSPERO; CRD420261348194) on 23rd March 2026.

We conducted a systematic review in line with the Preferred Reporting Items for Systematic Reviews and Meta-analyses (PRISMA) guidelines [16]. A systematic search was conducted using PubMed, MEDLINE, Embase, and Cochrane Library. Due to the rare nature of ENKTL, we searched for studies published from database inception to 19th March 2026. This ensured all the available literature on ocular and orbital involvement was collated. Two independent reviewers (RB and AF) screened titles and abstracts; disagreements were resolved by a third reviewer (DK). The search strategy combined keywords relevant to the study aim, and a sample strategy is shown in Table 1.

**Table 1:** Detailed search strategy for literature identification.

Database	Search terms
PubMed	("NK/T-cell lymphoma"[Title/Abstract] OR "extranodal NK/T-cell lymphoma"[Title/Abstract] OR ENKTL[Title/Abstract] OR "natural killer/T-cell lymphoma"[Title/Abstract]) AND (ocular[Title/Abstract] OR orbital[Title/Abstract] OR ophthalmic[Title/Abstract] OR eye[Title/Abstract] OR intraocular[Title/Abstract] OR lacrimal[Title/Abstract] OR uveitis[Title/Abstract] OR orbit[Title/Abstract] OR eyelid[Title/Abstract] OR conjunctival[Title/Abstract] OR "ocular adnexal"[Title/Abstract])

The search strategy was adapted for each database. In addition, reference lists of included studies were manually screened to identify any additional relevant articles.

Duplicate studies were removed, and the remaining studies were screened against predefined inclusion criteria for the review. In circumstances where there was uncertainty as to the relevance of the article, the full text of the paper was reviewed and scrutinised against the inclusion and exclusion criteria.

Studies were included if they met the following criteria: reported cases of extranodal NK/T-cell lymphoma with ocular or orbital involvement; included relevant clinical outcomes (e.g., survival, mortality, relapse, and visual outcomes); and reported prognostic factors with sufficient data to allow meaningful analysis. Only studies published in English were included. Eligible study designs were restricted to case reports, case series, cohort studies, and retrospective analyses. Review articles, editorials, and conference abstracts without full texts were excluded. These criteria are summarised in Table 2.

**Table 2:** Inclusion and exclusion criteria for study selection.

Inclusion criteria	Exclusion criteria
Studies reporting extranodal natural killer/T-cell lymphoma (ENKTL) with ocular or orbital involvement	Studies not specific to ENKTL
Studies reporting relevant clinical outcomes (e.g., survival, mortality, relapse, and visual outcomes)	Studies without ocular or orbital involvement
Studies reporting prognostic factors with sufficient clinical detail	Studies with poor or incomplete outcome reporting
Study designs including case reports, case series, cohort studies, and retrospective analyses	Review articles, editorials, and conference abstracts without full text
Studies published in English	Non-clinical studies or basic science studies

**Figure 1:**
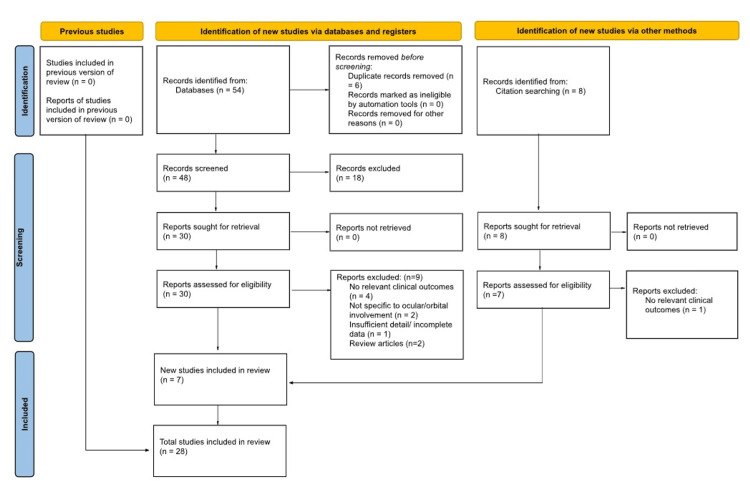
PRISMA diagram for the systematic review. Studies were excluded due to not being specific to extranodal natural killer/T-cell lymphoma, lack of orbital and ocular involvement, poor reporting of clinical outcomes, review articles without original patient data, non-clinical studies and studies focusing on other lymphoma subtypes (e.g., B-cell lymphomas).

Full text for all titles that appeared to meet the inclusion criteria was obtained. A PRISMA flowchart displaying the progress is provided in Figure 1, with reasons for studies being excluded from the systematic review stated in the figure legend.

Data extraction was performed independently using a standardised data collection framework. The extracted variable included patient demographics (number of patients, age, and sex). Disease characteristics included the site of involvement (orbital, intraocular, or adnexal), whether the disease was primary or secondary, the patient's EBV status, and disease stage, where available. We also reviewed the treatment variables, which were classified as chemotherapy, radiotherapy, surgical intervention, or stem cell transplantation.

Clinical outcomes were organised as primary and secondary. The primary outcome was overall survival. Secondary outcomes included mortality, disease progression or relapse, and visual outcomes. The prognostic factors evaluated included disease stage, EBV status, diagnostic delay, CNS involvement, and the extent of systemic disease.

Regarding quality assessment, it was recognised that there was a clear predominance of small-scale case reports and series. We assessed the quality of studies based on the confirmation of diagnosis, clinical data completeness, and clear reporting of treatment and outcomes. Greater weighting was given to larger cohort studies, extensive case series, and studies with a focus on reporting survival outcomes.

A quantitative meta-analysis was not performed due to the heterogeneity in study design, patient populations, and outcome reporting, particularly the variation in primary disease and secondary involvement, and the inconsistent outcome measures.

Results

Study Characteristics

A total of 28 studies were included in this systematic review. Of the included articles, the majority were single case reports (n = 20), this was followed by retrospective cohort analyses (n = 4), case series (n = 3), and population-based studies (n = 1), reflecting the rarity of ocular and orbital involvement in ENKTL.

Further details of study characteristics with patient demographics are summarised in Table 3, with treatment and clinical outcomes detailed in Table 4, and prognostic factors outlined in Table 5. Quality assessment of the studies using the Joanna Briggs Institute (JBI) criteria [17] has been outlined in Table 6. The results have been presented as a narrative synthesis, highlighting the key findings of the study in a qualitative form. This is due to the heterogeneity of the studies, resulting in significant variation in study design and outcome reporting.

**Table 3:** Summarised study characteristics including study title and publication date, study design, number of patients included in each article, patient age (median age calculated in larger studies), site of involvement (orbital, intraocular, or adnexal), and disease classification (primary or secondary). Mixed disease type refers to studies including both primary and secondary disease or where this distinction was not clearly reported.

Study	Year	Study design	N	Age (years)	Country	Site of involvement	Disease type
Dhodapkar et al. [2]	2023	Case report and systematic review	96	~40–70	USA	Orbital	Mixed
Hu et al. [6]	2019	Retrospective cohort	278	<60 = 80%	China	Nasal ± ocular	Secondary
Sukon et al. [7]	2022	Case report	1	43	Thailand	Intraocular	Primary
Papalkar et al. [8]	2005	Case report	1	41	India	Orbital	Secondary
Al Shawabkeh et al. [9]	2016	Case report	1	25	Jordan	Peri-orbital	Secondary
Luemsamran et al. [14]	2013	Case report	1	39	Thailand	Adnexal	Secondary
Loap et al. [18]	2024	Population-based study	9	67	USA	Mixed	Primary
Matsuo et al. [19]	2017	Case report	1	55	Japan	Intraocular	Secondary
Srimanan et al. [20]	2025	Case report	1	78	Thailand	Orbit + intraocular	Primary
Wei et al. [21]	2021	Case report	1	56	China	Intraocular	Primary
Widmer et al. [22]	2005	Case report	1	41	Switzerland	Conjunctiva	Primary
Okada et al. [23]	2018	Case report	1	73	Japan	Orbital + intraocular	Primary
Wong et al. [24]	2019	Case report	1	55	Hong Kong	Orbital	Secondary
Luo et al. [25]	2025	Case series	12	47	China	Intraocular	Mixed
Jones et al. [26]	2019	Case report	1	59	USA	Lacrimal sac	Secondary
Chen et al. [27]	2017	Case report	1	40	China	Orbital	Secondary
Ely et al. [28]	2012	Case report	1	22	USA	Orbital	Secondary
Türker et al. [29]	2012	Case report	1	57	Turkey	Orbital	Secondary
Kuwabara et al. [30]	2003	Case report	1	81	Japan	Orbital	Secondary
Anggraini et al. [31]	2021	Case series	2	45	Indonesia	Orbital	Secondary
Pupwe et al. [32]	2026	Case report	1	Young adult (~20s)	Zambia	Orbital	Primary
Mohapatra et al. [33]	2011	Case report	1	50	India	Orbital	Secondary
Yoo et al. [34]	2012	Case report	1	57	South Korea	Intraocular	Secondary
Zhang et al. [35]	2019	Case report	1	33	China	Intraocular	Secondary
Lee et al. [36]	2026	Case report	1	64	South Korea	Intraocular	Secondary
Jing et al. [37]	2022	Case report	1	61	China	Orbital	Primary
Han et al. [38]	2021	Case report	1	52	China	Nasal + ocular	Secondary
Li et al. [39]	2025	Case series	11	54	China	Orbital + periorbital	Mixed

**Table 4:** Treatment regimens and associated clinical outcomes. Data include the treatment type (chemotherapy, radiotherapy, immunotherapy); specific chemotherapy regimen has been included in brackets if available. Response to treatment, survival outcomes (with duration available), mortality outcomes, and disease progression or relapse were assessed, and the visual outcome with descriptive clinical terms in logMAR was not reported. Articles that did not have treatment-related data were not included in the table. Where information is not available in the full-text articles, NR (not reported) has been stated. Chemotherapy regimens are listed below: SMILE: steroid (dexamethasone), methotrexate, ifosfamide, L-asparaginase, etoposide; CHOP: cyclophosphamide, hydroxydaunorubicin (doxorubicin), oncovin (vincristine), prednisolone; CHOP-E: cyclophosphamide, epirubicin, vindesine, etoposide, prednisone acetate; DEVIL: dexamethasone, etoposide, vincristine, ifosfamide, L-asparaginase; GDP: gemcitabine, dexamethasone, platinum (usually cisplatin); B-FC: bendamustine, fludarabine, cyclophosphamide; GEMOX: gemcitabine, oxaliplatin, dexamethasone, pegaspargase. RT: radiotherapy; SCT: stem cell transplantation; RE: right eye; LE: left eye; HM: hand motion; NLP: no light perception; LP: light perception; KP: keratic precipitates.

Study	Treatment	Response	Survival	Mortality	Relapse/progression	Visual outcome
Dhodapkar et al. 2023 [2]	Chemotherapy ± RT	Temporary response	Median ~6 months	30%	Recurrence 34%; remission 34%	Visual acuity not quantifiable (reported impairment in 47%)
Sukon et al. 2022 [7]	Chemotherapy (CMT)	Initial improvement	2 years	Death	Progressive disease	Phthisis bulbi with no light perception
Papalkar et al. 2005 [8]	No treatment	No response	11 days	Death	Rapid progression	Visual acuity preserved (no logMAR change reported)
Al Shawabkeh et al. 2016 [9]	Chemotherapy (SMILE)	Response	NR	Survival	Complete remission	Stable visual acuity
Luemsamran et al. 2013 [14]	Chemotherapy (CHOP + Methotrexate)	Limited response	2 months	Death	Rapid progression	Vision loss with visual acuity not specified
Loap et al. 2024 [18]	Chemotherapy (88.8%), radiotherapy (66.6%)	NR	Median 15 months	66.6%	Disease progression 55.5%	NR
Srimanan et al. 2025 [20]	Chemotherapy (L-asparaginase + methotrexate)	Good response	NR	Survived	NR	Initial: 0.7 logMAR (RE), 0.5 logMAR (LE) → improved to 0.3 logMAR in both eyes → final deterioration to counting fingers (~1.6-2.0 logMAR)
Wei et al. 2021 [21]	Chemotherapy (SMILE)	Initial response	Relapse at 3 months	Death	CNS relapse	Rapid decline in visual acuity (final logMAR not reported)
Widmer et al. 2005 [22]	Chemotherapy + RT	Mixed response	Median 17 months	6/12 deaths	Majority progression	4/6 eyes >2.0 logMAR (worse than HM)
Okada et al. 2018 [23]	Chemotherapy (SMILE)	Response	14 months	Survived	Remission	LE restored to 0.0 logMAR
Luo et al. 2025 [25]	Chemotherapy ± RT	Partial response	Median 17 months	6/12 deaths	Disease progression	5/7 eyes >2.0 logMAR (worse than HM), 2 eyes 0.1 logMAR
Jones et al. 2019 [26]	Chemotherapy (SMILE) + Immunotherapy	Remission	NR	Survival	Remission	0.4 logMAR (RE), 0.2 logMAR (LE)
Chen et al. 2017 [27]	Chemotherapy (B-FC) + SCT	Response	NR	Survived	Relapse then remission	NR
Ely et al. 2012 [28]	Chemotherapy + RT	No sustained response	<3 months	Death	Rapid progression	Vision loss with visual acuity not specified
Türker et al. 2012 [29]	Chemotherapy (CHOP) + RT	Partial response	16 months	Death	Disease progression	Bilateral NLP (logMAR not defined)
Kuwabara et al. 2003 [30]	Radiotherapy	Initial response	4 months	Death	Metastatic progression	Progression to NLP (logMAR not defined)
Anggraini et al. 2021 [31]	Chemotherapy (DEVIL + CHOP)	Limited response	6 months	Death	Metastatic progression	0.3 logMAR (RE), 0.0 logMAR (LE)
Pupwe et al. 2025 [32]	Chemotherapy (GDP)	Initial response	Died during treatment	Death	Treatment-related death	Visual acuity not specified
Mohapatra et al. 2011 [33]	Chemotherapy (CHOP)	No response	Died during treatment	Death	Treatment-related death	LP (≥2.7 logMAR)
Yoo et al. 2012 [34]	Chemotherapy (Triamcinolone) + RT	Improvement	3 months	Death	Disease progression	Visual acuity of the affected eye with correction was 0.0/0.2 logMAR
Zhang et al. 2019 [35]	Chemotherapy (L-asparaginase)	Limited response	1 month	Death	Disease progression	Light perception (LP; approx. ≥2.7 logMAR)
Lee et al. 2026 [36]	SMILE	Complete remission	8 months	Death	Relapse	Cataract development, KP nearly resolved.
Jing et al. 2022 [37]	GEMOX initially and CHOP-E + RT	Complete remission	Survived	N/A	Nil	Corrected visual acuity to 20/20, full eyeball movement
Han et al. 2021 [38]	SMILE + intravitreal methotrexate	Improvement	Survived	N/A	Initial recurrence from the nasal primary	Symptoms improved; however, exudate and retinal detachment remained
Li et al. 2025 [39]	Chemotherapy + RT	Various	5 died, 5 lost to follow-up	1 survived	Various	

**Table 5:** Prognostic factors and clinical outcomes. The factor category provides a brief overview of the prognostic factor, with further detail provided in the neighbouring column. Only studies reporting associations between prognosis and outcomes were included. Findings are summarised qualitatively due to the heterogeneity in study design and outcome reporting. EBV: Epstein- Barr virus; OS: overall survival.

Study	Factor category	Prognostic factor	Associated clinical outcome
Dhodapkar et al. 2023 [2]	Diagnostic/treatment	Misdiagnosis, relapse after initial response, orbital involvement	Delayed diagnosis (57.5%), high relapse rates, poor survival
Sukon et al. 2022 [7]	Diagnostic	Delayed diagnosis	Advanced disease at presentation and poorer outcomes
Papalkar et al. 2005 [8]	Treatment/temporal	Chemotherapy resistance, rapid progression	Early death (within days to weeks)
Loap et al. 2024 [18]	Disease-related	Advanced stage (III/IV), advanced age	Reduced overall survival (median OS of 15 months), increased mortality
Wei et al. 2021 [21]	Disease-related	CNS involvement	Early relapse, rapid disease progression, poor survival
Widmer et al. 2005 [22]	Disease-related/systemic	EBV positivity, bone marrow involvement	Aggressive disease, rapid deterioration, death
Luo et al. 2025 [25]	Disease-related	Advanced stage, multisystem disease	Increased mortality and disease progression
Ely et al. 2012 [28]	Diagnostic/temporal	Misdiagnosis, rapid progression	Severe disease progression and early mortality (<3 months)
Türker et al. 2012 [29]	Ocular	Bilateral severe visual loss	Advanced disease and poor outcomes
Anggraini et al. 2021 [31]	Treatment/systemic	Treatment-related complications (sepsis)	Early mortality
Pupwe et al. 2025 [32]	Treatment	Treatment-related toxicity	Death during treatment
Mohapatra et al. 2011 [33]	Treatment	Chemotherapy resistance	Disease progression and mortality
Zhang et al. 2019 [35]	Ocular	Severe visual loss at presentation	A marker of advanced disease and poor prognosis
Jing et al. 2022 [37]	Diagnostic	Delayed diagnosis	Advanced disease
Li et al. 2025 [39]	Disease-related and diagnostic	Advanced stage and delayed diagnosis	Early mortality

**Table 6:** Study quality assessed using the Joanna Briggs Institute (JBI) critical appraisal checklists for case reports and case series. Studies were evaluated across domains, including patient description, diagnostic confirmation, intervention reporting, outcome reporting, and adequacy of follow-up. Each domain was scored as ‘yes’ or ‘no’, and an overall score (0-5) was calculated. Studies scoring 5 were classified as low risk of bias, scores of 3-4 as moderate risk, and ≤2 as high risk. Overall study quality was variable, with most studies demonstrating low to moderate risk of bias, while several case reports showed greater concern due to limited follow-up and incomplete reporting.

Study	Patient description	Diagnosis	Intervention	Outcomes	Follow-up	Score	Overall risk
Dhodapkar et al. 2023 [2]	Yes	Yes	Yes	Yes	Yes	5	Low
Hu et al. 2019 [6]	Yes	Yes	Yes	Yes	No	4	Moderate
Sukon et al. 2022 [7]	Yes	Yes	Yes	Yes	No	4	Moderate
Papalkar et al. 2005 [8]	Yes	Yes	No	Yes	No	3	Moderate
Al Shawabkeh et al. 2016 [9]	Yes	Yes	No	Yes	No	3	Moderate–High
Luemsamran et al. 2013 [14]	Yes	Yes	Yes	Yes	Yes	5	Low
Loap et al. 2024 [18]	Yes	Yes	Yes	Yes	No	4	Moderate
Matsuo et al. 2017 [19]	Yes	Yes	Yes	Yes	No	4	Moderate
Srimanan et al. 2025 [20]	Yes	Yes	Yes	Yes	No	4	Moderate
Wei et al. 2021 [21]	Yes	Yes	Yes	Yes	No	4	Moderate
Widmer et al. 2005 [22]	Yes	Yes	Yes	Yes	Yes	5	Low
Okada et al. 2018 [23]	Yes	Yes	Yes	Yes	Yes	5	Low
Wong et al. 2019 [24]	Yes	No	No	No	No	1	High
Luo et al. 2025 [25]	Yes	Yes	Yes	Yes	Yes	5	Low
Jones et al. 2019 [26]	Yes	Yes	No	Yes	No	3	Moderate
Chen et al. 2017 [27]	Yes	Yes	Yes	Yes	No	4	Moderate
Ely et al. 2012 [28]	Yes	Yes	Yes	Yes	Yes	5	Low
Türker et al. 2012 [29]	Yes	Yes	Yes	Yes	No	4	Moderate
Kuwabara et al. 2003 [30]	Yes	Yes	No	Yes	No	3	Moderate
Anggraini et al. 2021 [31]	Yes	Yes	Yes	Yes	No	4	Moderate
Mohapatra et al. 2011 [33]	Yes	No	No	Yes	No	2	High
Yoo et al. 2012 [34]	Yes	Yes	Yes	Yes	No	4	Moderate
Zhang et al. 2019 [35]	Yes	Yes	No	Yes	No	3	Moderate
Lee et al. 2026 [36]	Yes	Yes	Yes	Yes	No	4	Moderate
Jing et al. 2022 [37]	Yes	Yes	Yes	Yes	Yes	5	Low
Han et al. 2021 [38]	Yes	Yes	Yes	Yes	Yes	5	Low
Li et al. 2025 [39]	Yes	Yes	Yes	Yes	Yes	5	Low

The included studies represented a broad geographical distribution, with most cohorts from East Asia (China, South Korea, and Japan), reflecting the higher prevalence of ENKTL in Asian populations. Additional studies were identified from South and Southeast Asia (India and Thailand), Europe (the United Kingdom and Germany), North America (the United States), and Africa (Zambia).

Across all studies, approximately 429 cases with ocular or orbital involvement of ENKTL were identified. Larger cohort and population-based studies contributed the majority of patients, whereas individual case reports comprised most of the included study designs, providing detailed insights into clinical presentation, presenting symptoms, choice of treatment, response, and disease progression.

Baseline Characteristics

Patient demographics were reported variably across studies, with many studies providing only summary-level details, which limited direct comparison. Patient age ranged from 22 to 81 years, with a median age of presentation of 53 years. Orbital involvement was the most reported site, with intraocular involvement also frequently reported. Several studies [18-20,40] reported mixed anatomical involvement, suggesting advanced disease or rapid disease progression at the time of diagnosis. Primary, secondary, and mixed diseases were all reported in the studies, with secondary disease as expected, predominating in the larger cohort studies. Primary disease was reported in a total of eight studies [7,20-24,37,38], highlighting its rarity. Mixed disease was reported in cases where the distinction between primary and secondary involvement was unclear.

Treatment Outcomes

Treatment and clinical outcomes were reviewed across 28 studies; four studies were not included in this synthesis as they had incomplete outcome reporting. Hu et al. (2019) did not include survival, mortality, or relapse data with a larger focus on nasal ENKTL [6]. Similarly, Wong et al. and Lee et al. reported very limited outcome data with no clear treatment-outcome relationship [24,36]. Matsuo et al. did not establish any detailed survival data, with only descriptive data of the case available [19]. For the remaining studies, data were collected across both survival and treatment outcomes. This included data on treatment and, where possible, the regimen the patient completed; survival times were measured in months, and mortality was measured as either death or, in larger studies, as the percentage of patients who died. This was the case in Loap et al., Matsuo et al., and Luo et al. [18,19,25]. Relapse and disease progression data were also collected to review disease-free survival. Lastly, we looked at the visual outcomes. These data were harder to standardise, but where possible, were standardised to logMAR results.

The treatment recorded was mainly chemotherapy. The most common regimens were non-anthracycline-based, which often preceded or were combined with radiotherapy treatment. There were two studies that did not fall into either receiving chemotherapy or radiotherapy. One study by Jones et al. reported treatment with immunotherapy [26], and a single case report by Chen et al. noted treatment with stem cell transplant alongside chemotherapy [27].

Looking at our results in larger cohort studies [6,18,25,39], chemotherapy was not consistently reported with the specification of treatment received; it is likely that it reflects a mixture of historical anthracycline-based therapies, such as CHOP (cyclophosphamide, doxorubicin, vincristine, prednisone) and contemporary asparaginase-containing therapies. Among the smaller case studies, the treatment regimen was more clearly stated and demonstrated the use of both anthracycline and non-anthracycline regimens. Although mortality remained high across all treatment approaches, cohort data reported mortality rates of 30-66.6% and median survival ranging from six to 17 months. Amongst individual cases, poorer outcomes were more frequently observed in patients treated with anthracycline-based or non-specific regimens. From a total of 20 single case reports and one case series, reports of surviving patients were eight; of these eight patients, six received non-anthracycline therapy, and the remaining patient in Chen et al.'s study [27] initially received SMILE (steroid, methotrexate, ifosfamide, L-asparaginase, etoposide) therapy, but this treatment was replaced by bortezomib, fludarabine, and cyclophosphamide (B-FC) as a salvage strategy due to disease relapse. Chen et al. describe a novel treatment using B-FC as an alternative in a case of relapsed ENKTL, achieving a successful result. Historically, B-FC has been used to treat chronic lymphocytic leukaemia, but for ENKTL, it is not a standard regimen and has only been reported in limited or experimental settings.

Radiotherapy was mainly used in combination with chemotherapy rather than as a standalone treatment option [2,22,25,28,29,34,37]. In the single case where radiotherapy was used as a standalone treatment, it yielded a poor outcome, with rapid disease progression and early mortality [30]. Across all studies using simultaneous therapy, disease progression was frequently recorded in the larger cohort studies [22,28,29], and mortality remained high, ranging from 30% to 66.6%. Visual outcomes also remained poor with studies reporting progression to severe visual impairment or blindness [28-30]. The findings suggest that while radiotherapy may provide benefit to local disease control, it is insufficient as monotherapy due to the aggressive nature of ENKTL and the high rate of relapse.

Initial treatment response varied among the studies, although many studies reported initial improvement to treatment; these responses were not sustained, and a high proportion of patients experienced further disease progression [8,22-25,28,38] or relapse [2,19,21,27]. Sustained remission was uncommon and limited to only a few isolated cases [9,23,26,27,37,38], predominantly followed by non-anthracycline or asparaginase-based therapies. CNS involvement and metastatic spread were reported in numerous cases and were linked to high rates of mortality [21,23].

The overall survival outcomes were poor, with median survival ranging from six to 17 months in larger series, with multiple studies describing rapid clinical deterioration often within weeks to months of initial diagnosis [8,21-23,28]. The most common causes of death were metastatic disease, leading to rapid progression as reported across multiple studies [2,7-9,18,22,23,25,28-30]. Relapse-associated deaths were also observed, particularly in patients with CNS involvement [2,19,21,25]. Sepsis or infection-related deterioration was reported in a small number of cases, likely secondary to advanced disease or treatment-related immunosuppression [34,35,38]. There were three clearly documented drug-related deaths, one due to a fatal case of hepatorenal failure following chemotherapy [32] and two further cases in immunocompromised patients [31-33]. Poor outcomes associated with anthracycline-based regimens were observed in several cases [29,33,35], supporting the limited efficacy of use in ENKTL. The remainder of the studies either had very limited mortality details [6,24,36] or the patients survived [20,26,27].

Visual outcomes were also reviewed, although due to the heterogeneity of reporting methods, it was difficult to standardise results. Where possible, results were converted to logMAR for direct comparison across studies. Overall visual outcomes were very poor, with most reports describing significant and rapid visual deterioration, quickly leading to death or permanent visual loss. Several studies reported patients progressing to severe visual impairment [7,28-30,34,35]. In larger cohort studies, visual outcomes were similarly poor. Luo et al. described the majority of patients in the review with visual acuity worse than 2.0 logMAR, corresponding to profound visual loss [25].

Prognostic Factors

Prognostic factors were variably reported across the included studies, with reports providing differing levels of detail regarding the factors influencing outcomes. The most notable prognostic factor leading to poorer outcomes was delayed diagnosis or advanced disease at the point of diagnosis. This often led to a quicker clinical deterioration and poor response to treatment [18,25]. The main cause of delayed treatment was due to misdiagnosis. ENKTL commonly masquerades as other common inflammatory and infectious pathologies, which led to steroid treatment being given in place of chemotherapy [8,9,31,34,38,39].

In terms of treatment factors influencing outcomes, the most critical factor was the use of anthracycline-based regimes. These were associated with poorer outcomes and survival as ENKTL is inherently non-responsive to these treatments [4,11]. Non-anthracycline regimes were associated with better outcomes and improved disease control and length of survival [20,21,23,26,27] but did not always improve mortality [21,35].

EBV association is an important factor to consider when reviewing prognostic influence, as it is a well-recognised feature of extra-nodal ENKTL. From the current literature, EBV-positive tumours have been shown to be associated with a more aggressive clinical phenotype, due to angioinvasion, rapid proliferation, and a higher likelihood of systemic dissemination [5,12,41]. The outcomes are further affected by a high viral load or widespread EBV involvement.

EBV status was very poorly reported across studies, representing a notable limitation given its established role in current literature. The EBV-driven oncogenesis has been associated with advanced disease and high rates of progression, and may explain the limited response to anthracycline-based chemotherapy compared to asparaginase-based regimes [4,5,11,12]. This is in the context of a limited and heterogeneous evidence base.

Discussion

Key Findings

ENKTL outcomes are strongly influenced by certain prognostic factors, although survival outcomes are generally poor, mortality rates and survival length can be improved by earlier diagnosis, avoiding misdiagnosis, and commencing treatment with asparaginase treatment regimes. However, the efficacy of asparaginase treatment regimens needs to be again appreciated within the context of a limited and heterogeneous evidence base.

To our knowledge, this is the first systematic review appraising prognostic factors in ocular and orbital involvement of extranodal ENKTLs. Previous studies, including Dhodapkar et al. [2], Hu et al. [6], Tse et al. [4], and Mohapatra et al. [33], have all had a broad focus around clinical presentation, diagnostic challenges, and treatment strategies, without a structured evaluation of prognostic determinants. Our review expands on the current literature available by systematically analysing the factors that influence the poor outcomes in this disease, mainly misdiagnosis, delay in treatment, and the delivery of resistant drugs. This provides a deeper understanding of disease progression and survival, highlighting areas that have been underreported in prior studies.

Diagnostic Delay

Delays in diagnosis and misdiagnosis were consistently associated with poor outcomes, including mortality. ENKTL closely mimics inflammatory conditions such as uveitis, orbital inflammation, and cellulitis. Papalkar et al. [8] describe a case that was ‘treated as idiopathic orbital inflammation with corticosteroids’ prior to fatal clinical deterioration. Anggraini et al. [31] reported cases ‘masquerading inflammatory processes’ lead to delayed diagnosis, and therefore, in the context of such an aggressive disease, lead to rapid deterioration. Similarly, Al Shawabkeh et al. [9] described treatment of presumed ‘peri-orbital cellulitis’ with corticosteroids for a sustained period, missing the ability to treat ENKTL before malignant spread. Yoo et al. [34] reported a case mimicking uveitis with again misdirected treatment.

There was a pattern in many of these cases presenting at an advanced stage, with several studies reporting multi-site involvement, CNS dissemination, and rapid disease progression at the time of correct diagnosis [21,23,29]. This then resulted in the majority of cases experiencing poor outcomes despite subsequent treatment. These cases emphasised the need for rapid diagnosis with a low threshold to investigate treatment-resistant ocular presentations or atypical presentations with early biopsy.

Resistance to Treatment

Resistance to treatment remains the leading cause of poor clinical outcomes in ENKTL. There is no standard treatment regimen as ENKTL is variable and highly dependent on both the stage and site of disease; however, there are agreed principles of treatment that influence management plans. Tse et al. [4] outline that this contemporary approach is based on NK cells expressing high levels of P-glycoprotein, leading to a multidrug resistance (MDR) phenotype, making it particularly difficult to treat. Previously, these lymphomas were treated with anthracycline-containing regimens such as CHOP, designed mainly for B-cell lymphomas; however, in ENKTLs, these regimens have shown limited effectiveness.

Non-anthracycline-containing regimens have since then been developed. The core component of these regimens is asparaginase, which has been shown to induce apoptosis of NK cells in vitro and has demonstrated clinical efficacy in relapsed or refractory ENKTL. Although many current case reports describe treatment with asparaginase-based treatments, treatment resistance persists, and particularly in cases of relapsing disease, there is a continuing need for further optimisation of therapeutic strategies [40].

Clinical Implications

From a clinical perspective, there is great importance in early recognition of ocular and orbital ENKTL to improve outcomes. Clinicians should have a high suspicion of atypical and refractory inflammatory eye conditions of standard therapy. Early tissue biopsy is essential to provide a definitive diagnosis to ensure treatment is commenced with immediate effect. Empirical corticosteroid treatment should be avoided where diagnosis is possible, as this may mask underlying disease and delay appropriate investigation and contribute to further disease progression. These findings have been supported by multiple case reports that demonstrate both delayed diagnosis and initial misdiagnosis, which led to either advanced disease at presentation or poor clinical outcomes [8,9,21,23,29,31,34].

Limitations

This systematic review has several limitations that should be considered when interpreting the findings. One of the main limitations is that the majority of the studies were single case reports, which resulted in a small sample size and introduced potential publication bias as more severe or atypical cases were included in the analysis. This limits the extent to which these findings relate to the wider population. In addition, there was significant heterogeneity across the included studies, with wide variation in reported population demographics, clinical presentations, treatment regimens, and style of outcome reporting. This variability limited direct comparison between studies and did not allow for quantitative synthesis of the results.

In addition to this, the quality of reporting across the studies was variable, with incomplete clinical and outcome data in several categories. This was highlighted in the reporting of prognostic factors, where narrative synthesis was performed to account for heterogeneity, which leaves the risk that findings may be swayed by reporting bias and incomplete capturing of the data.

Future Directions

This review highlights the need for further research, particularly multicentre studies and registries; however, this may be difficult given the rare nature of the disease. It is possible that the true incidence of ENKTL is underestimated, as patients who are initially misdiagnosed may experience fatal outcomes before a definitive diagnosis is established, potentially leading to underreporting of cases in the literature. Therefore, it is important to collect collaborative data to improve understanding of the disease behaviour, treatment response, and long-term outcomes.

In addition, there is a need for standardised reporting across studies, including consistency of variables such as EBV status, disease stage, treatment regimes, and clinical outcomes, including visual outcomes.

Standardisation would allow direct comparability between studies and allow for a more robust synthesis of data in future analysis.

Further research is also required to evaluate optimal treatment strategies, particularly the role of asparaginase regimes and therapies to deliver in relapsing disease. These robust treatment regimes are needed to improve survival outcomes in this aggressive disease.

## Conclusions

Ocular and orbital involvement in ENKTL is rare but associated with a poor prognosis and high mortality. Delayed diagnosis and advanced disease at presentation are the salient causes of adverse outcomes. This is most often driven by the diseases masquerading as common inflammatory conditions. Early diagnosis, prompt biopsy, and timely treatment are essential for improving survival outcomes. Some thought can be given to the appropriate use of non-anthracycline-based treatment. Further research with larger cohorts and standardised reporting is required to improve treatment strategies for this aggressive disease.
